# Nephrotoxicity in the Age of Immune Checkpoint Inhibitors: Mechanisms, Diagnosis, and Management

**DOI:** 10.3390/ijms25010414

**Published:** 2023-12-28

**Authors:** Krishna Moturi, Harsh Sharma, Neda Hashemi-Sadraei

**Affiliations:** Department of Medicine, Division of Hematology and Oncology, University of New Mexico Comprehensive Cancer Center, Albuquerque, NM 87131, USA; hrsharma@salud.unm.edu

**Keywords:** immune checkpoint inhibitors (ICI), acute kidney injury (AKI), ICI-related nephritis, management of ICI-AKI, nephritis as a predictor of treatment response, biomarkers of ICI-AKI, renal biopsy, renal complications, auto-antibodies, auto-reactive T cells, drug-specific T cells

## Abstract

Immune checkpoint inhibitors (ICI) revolutionized cancer therapy by augmenting anti-tumor immunity via cytotoxic T-lymphocyte-associated protein 4 (CTLA-4) and programmed death-1/programmed death-ligand 1 (PD-1/PD-L1). However, this breakthrough is accompanied by immune-related adverse effects (irAEs), including renal complications. ICI-related nephritis involves complex mechanisms like auto-reactive T cells, auto-antibodies, reactivation of drug-specific T cells, and cytokine-driven inflammation culminating in AKI. ICI-AKI typically manifests weeks to months into treatment, often with other irAEs. Timely detection relies on monitoring creatinine levels and urine characteristics. Biomarkers, like soluble interleukin-2 receptor (sIL-2R) and urine cytokine levels, provide non-invasive insights, while renal biopsy remains the gold standard for confirmation. Management of ICI-AKI requires a balance between discontinuing ICI therapy and prompt immunosuppressive intervention, typically with corticosteroids. Some cases permit ICI therapy resumption, but varying renal recovery rates highlight the importance of vigilant monitoring and effective therapy. Beyond its clinical implications, the potential of irAEs to predict positive treatment responses in certain cancers raises intriguing questions. Data on nephritis–treatment response links are limited, and ongoing research explores this complex interaction. In summary, ICI therapy’s transformative impact on cancer treatment is counterbalanced by irAEs, including nephritis. Early recognition and management are vital, with ongoing research refining diagnostic and treatment strategies.

## 1. Introduction

Over the past decade, the field of cancer therapy has witnessed a profound and transformative evolution, chiefly driven by the emergence of immune checkpoint inhibitors (ICIs). Among the extensively scrutinized ICIs are cytotoxic T-lymphocyte antigen 4 (CTLA-4), programmed cell death 1 (PD-1), and programmed cell death ligand 1 (PD-L1) inhibitors, which have effectively reshaped the treatment landscape for a spectrum of malignancies.

Cancer cells inherently present antigens that are susceptible to recognition by the immune system. Nevertheless, they have developed a repertoire of mechanisms to elude immune detection and destruction. One such strategy exploits negative feedback through the engagement of inhibitory receptors like CTLA-4 and PD-1. CTLA-4 finds its expression on CD4-positive and CD8-positive lymphocytes and engages co-stimulatory factors on antigen-presenting cells (APCs). This interaction exerts a suppressive influence, resulting in diminished production of Interleukin-2 (IL-2) and, consequently, a reduction in T cell proliferation. Conversely, PD-1 is expressed on multiple immune cell types, including T cells, B cells, and NK cells. One of its ligands, PD-L1, is prevalent across diverse cell types, including tumor cells, and fulfills a role in inhibiting previously activated T cells [1].

The introduction of immune checkpoint inhibitors (ICIs) effectively counteracts the immune evasion orchestrated by tumor cells, fostering a pro-inflammatory tumor microenvironment that holds the potential to enhance disease control. However, this advancement comes with a caveat—a heightened risk of inflammatory-mediated toxicities. While immune-related adverse events (irAEs) affecting the gastrointestinal and skin organ systems are more common, renal irAEs can also occur, posing a distinct challenge in the management of ICI therapy. Renal irAEs typically manifest as acute kidney injury (AKI), which is defined as an increase in serum creatinine of ≥0.3 mg/dL or ≥1.5 times the baseline value according to the Kidney Disease: Improving Global Outcomes (KDIGO) criteria [2]. The reported incidence of ICI-induced AKI varies widely across the available literature. Notably, the combination of CTLA4 and PD1 inhibitors has been associated with a higher incidence of ICI-related nephritis (4%) compared to monotherapy (approximately 2%) [3]. Recent systematic reviews and meta-analyses have provided insights into the prevalence of AKI in the context of ICI therapy, reporting an overall incidence of around 16.0%, with 3.5% attributed to ICI-induced AKI [4]. Furthermore, a meta-analysis found the pooled relative risk of AKI in patients treated with PD-1 inhibitors compared to non-nephrotoxic therapies to be 4.19% [5].

While the absolute incidence of ICI-AKI may not be exceedingly high, its consequences can be profound. It can lead to the permanent discontinuation of ICI treatment, the development of chronic renal dysfunction, and an increased risk of mortality. In fact, patients who develop ICI-AKI face a 51% elevated risk of mortality compared to those without AKI [4]. Consequently, the precise mechanisms and optimal management of ICI-induced nephritis remain active areas of research, underscoring the importance of vigilant monitoring and effective intervention strategies to mitigate its impact in the context of ICI therapy.

The relevance of ICI-related nephritis becomes even more pronounced when considering that a substantial proportion of patients undergoing ICI treatments have previously been exposed to other potentially nephrotoxic agents, such as chemotherapy. This cumulative nephrotoxic burden raises concerns about the kidney’s compensatory capacity in the face of ICI-mediated renal damage.

## 2. Mechanisms/Molecular Basis of ICI-Related Nephritis

The precise etiology of ICI-related nephritis is not known, and the various postulated mechanisms are based on pre-clinical data. Proposed mechanisms associated with ICI-related nephritis include auto-reactive T cells and loss of tolerance to self-antigens; auto-antibodies; checkpoint receptor expression on non-tumor tissue; reactivation of drug-specific T cells; and cytokines and inflammation. The subsequent discussion will delve into the individual contributions of each of these factors in the development of AKI. 

### 2.1. Auto-Reactive T Cells and Loss of Self-Tolerance

**Figure 1:**
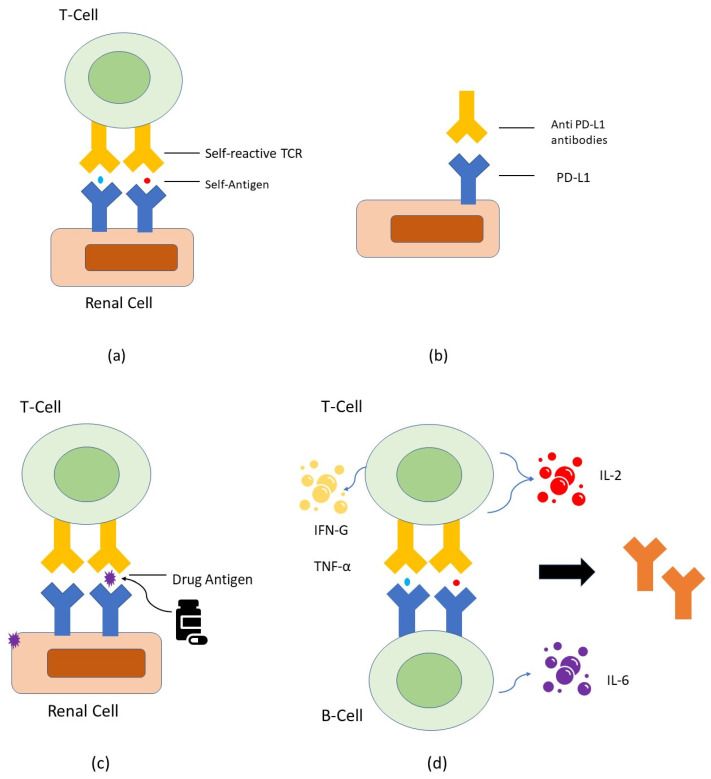
(**a**) Schematic diagram depicting auto-reactive T cells and loss of self-tolerance to antigens. Self-reactive T cells that exit from the thymus are normally kept dormant through various mechanisms including CTLA-4 and PD-1 checkpoint signaling. Removal of checkpoint signaling in the context of ICI therapy may lead to the activation of self-reactive T cells and kidney damage; (**b**) Schematic diagram depicting checkpoint receptor expression on non-tumor tissue. PD-L1 is expressed at low levels on renal tubular cells and can be significantly upregulated by interferon treatment. Anti-PD-L1 antibodies can theoretically bind to these cells and cause renal injury; (**c**) Schematic diagram depicting reactivation of drug-specific T cells. Patients who are taking medications that can cause tubular injury (like PPIs, NSAIDs, or antibiotics) can directly or indirectly trigger an immune response by binding to tubular antigens and acting as haptens. These haptens can get trapped in the kidney tissue and lead to tubular damage. Auto-reactive T cells, which may be dormant initially, could become activated when ICIs are administered; (**d**) Schematic diagram depicting development of auto-antibodies. ICI administration could result in the production of auto-antibodies directed against antigens presented by TECs, mesangial cells, or podocytes.

One possible mechanism is the development and proliferation of auto-reactive T cells. This was seen in some cases of ICI-induced myocarditis, where selective clonal T cell populations were noted to be infiltrating the myocardium [6]. Murine models have shown that PD-1 signalling is essential to the tolerance of self-antigens. PD-1 signalling limits CD-8-positive T-cell-mediated inflammatory injury and PD-1 knockout mice spontaneously develop glomerulonephritis [7,8]. Thus, PD-1 inhibition may drive an autoimmune variant of interstitial nephritis (Figure 1a).

### 2.2. Auto-Antibodies

Another postulated mechanism is the development of auto-antibodies against antigens that are present on tubular epithelial cells, mesangial cells, and podocytes. In support of this mechanism, patients treated with ipilimumab developed nephrotic syndrome and a lupus-like immune-complex glomerulopathy [9,10]. These patients were found to have anti-CTLA4 antibodies, anti-double-stranded DNA (anti-dsDNA) antibodies, and anti-nuclear antigen (ANA) antibodies. The interruption of ICI and steroids decreased the antibody levels. 

### 2.3. Checkpoint Receptor Expression on Non-Tumor Tissue

An alternative hypothesis is the expression of checkpoint receptors on non-tumor tissues. PD-L1 is expressed at low levels on renal tubular cells and can be significantly upregulated by interferon treatment [8]. Anti-PD-L1 antibodies can theoretically bind to these cells and cause renal injury. Additionally, regulatory T cells (Tregs) protect the kidney from ischemia–reperfusion injury, and the blockade of PD-1 on Tregs was found to negate their ability to protect against kidney injury [11] (Figure 1b).

### 2.4. Reactivation of Drug-Specific T Cells

Another possible mechanism is the ICI-induced reactivation of drug-specific T cells. Patients who received medications, such as proton pump inhibitors (PPIs), nonsteroidal anti-inflammatory drugs (NSAIDs), or antibiotics that are commonly associated with acute tubulointerstitial nephritis (ATIN), can trigger an immune response by acting as haptens [12]. It was postulated that drug-specific T cells, which are generally inhibited by PD-1 signalling, are instead activated when ICIs are administered and lead to an aberrant immune response and tubular damage [13]. In support of this hypothesis, there were reports of patients who developed ICI-induced ATIN when concurrently receiving PPIs or NSAIDs. They experienced renal recovery when the concomitant drug was stopped, suggesting that the T-cell creativity was directed to the medication rather than a self-antigen [14] (Figure 1c).

### 2.5. Cytokines and Inflammation

Finally, the administration of ICIs can cause inflammatory renal injury through increased production and elaboration of pro-inflammatory cytokines. IL-10 levels were found to be related to a higher risk of development of irAE in lung cancer patients [15] (Figure 1d).

## 3. Diagnosis

### 3.1. Clinical Presentation

In individuals with cancer, AKI predominantly stems from pre-renal factors, like volume depletion or sepsis. Therefore, conducting a comprehensive patient history and a meticulous physical examination to assess and address the common culprits of AKI is imperative. This approach not only aids in early diagnosis and appropriate management but also helps to circumvent the need for costly and extensive diagnostic evaluations. 

Several extensive multicenter investigations have undertaken the scrutiny of the clinical features associated with ICI-AKI [13,14]. Typically, the onset of AKI is observed at a median of 14–16 weeks following the initiation of ICIs, with manifestations occurring within 2–3 weeks after the last administered dose. It is noteworthy that nearly half of the patients manifest prior or concurrent extrarenal irAEs, with common clinical presentations encompassing cutaneous manifestations such as rash and hepatic disorders such as hepatitis.

### 3.2. Risk Factors

Understanding the risk factors associated with ICI-induced AKI is pivotal for effective management and early intervention. Through extensive clinical studies and meta-analyses, several key factors have emerged:

- **Age and sex:** Although there are limited data, there appeared to be no notable differences observed with regard to advancing age, male gender, white race, or other pre-existing comorbidities [4,16].
- **Drugs:** Renal irAEs are potentially more prevalent in patients undergoing ICI therapy combined with PPIs, NSAIDs, or antibiotics [4,17]. Combining ICIs with these medications, particularly NSAIDs and PPIs, has been linked to AIN, potentially activating drug-specific T cells, and triggering immune reactions. While some studies showed no significant NSAID-AKI association [3,18], systematic reviews identified NSAIDs as risk factors [19]. Additionally, drugs like fluindione [19] have also been associated with increased AKI risk during ICI therapy. The use of PPIs also showed a potential link [18,19], although cautious interpretation is necessary due to the lack of evidence from randomized controlled trials, as confounding factors might influence the results.
- **Malignancies and ICI classes:** Genitourinary cancers were associated with a higher AKI risk in ICI-treated patients, whereas lung cancer and melanoma demonstrated varying risks [20]. It is essential to acknowledge that these data encompassed both ICI-AKI and non-ICI-AKI cases. None of the ICI categories (anti-PD-1, anti-PD-L1, or anti-CTLA-4) showed a distinct propensity for AKI. However, combining anti-PD-1/PD-L1 with anti-CTLA-4 significantly correlated with AKI [18,20].
- **Chemotherapy:** Limited data exist regarding patients with prior chemotherapy exposure as a risk factor for AKI. In a single study addressing this, the risk of sustained AKI in patients with a history of nephrotoxic chemotherapy did not achieve statistical significance (adjusted hazard ratio (aHR), 1.52; 95% confidence interval (CI), 0.95 to 2.44; *p* = 0.08) [3]. A pharmacovigilance study based on the US Food and Drug Administration (FDA) Adverse Event Reporting System (FAERS) database between January 2014 and June 2019 revealed that ICIs plus chemotherapy strategies reported more renal toxicities compared to sole ICI regimens (ROR: 1.30, 95% CI: 1.17–1.45) [21]. However, it is important to note that the study does not distinguish whether these renal toxicities are specifically attributable to ICI-associated nephrotoxicity (ICI-AKI) or stem from other etiologies.
- **Baseline chronic kidney disease (CKD):** pre-existing CKD has been identified as a risk factor for ICI-induced AKI [1,17,18].
- **Other irAE:** The development of non-kidney irAEs was associated with an increased risk of renal irAE [4,17]. This is likely due to heightened immune system activation, potentially contributing to off-target immune effects within the kidney.

Understanding these risk factors is critical for identifying high-risk patients undergoing ICI therapy. Future research, incorporating larger study cohorts, is essential to unravel the intricate complexities of these risk factors and their interplay in the context of ICI therapy.

### 3.3. Laboratory Findings

Monitoring serum creatinine is often the primary indicator in most cases, and it serves as a pivotal parameter in the vigilant oversight of patients. Regular and meticulous assessment of serum creatinine levels before each treatment cycle is imperative. It is important to note that a rise in serum creatinine is commonly observed following significant damage to the entire nephron mass, and therefore creatinine is not an ideal early biomarker for renal damage. However, a comprehensive diagnostic approach necessitates a more thorough evaluation.

In addition to serum creatinine, the inclusion of urine chemistries and urinalysis is strongly recommended. This comprehensive urinalysis should encompass the examination of urine sediment and the analysis of critical parameters such as spot urine albumin–creatinine ratio (UACR) and urine protein–creatinine ratio (UPCR). The presence of a high UACR should raise suspicion of ICI-associated glomerular disease and lower the clinical threshold for proceeding with a kidney biopsy.

Furthermore, the analysis of urine chemistries, including the calculations of fractional excretion of sodium (FeNa) and fractional excretion of urea (FeUrea), can be instrumental in distinguishing between pre-renal AKI and acute tubular necrosis (ATN). These parameters aid in refining the diagnostic process and guiding appropriate interventions.

It is worth noting that urinary abnormalities, such as the presence of pyuria, proteinuria, and eosinophilia, have been observed in patients with ICI-related nephritis. While these abnormalities are nonspecific and cannot solely differentiate AKI attributable to ICI therapy from other potential etiologies, they should raise suspicion and prompt a more thorough evaluation for ICI-induced AKI. 

### 3.4. Imaging

In a retrospective analysis conducted at a single medical center, the imaging features of ICI-related nephritis were systematically assessed using computed tomography (CT) and positron emission tomography with CT (PET-CT) [22]. This meticulous examination revealed distinct radiological findings indicative of the condition. Notably, a bilateral increase in kidney volume, the emergence of new or progressively enhancing perinephric fat stranding, and the development of hypo-enhancing wedge-shaped cortical foci were identified as characteristic features in cases of ICI-related nephritis. Furthermore, it was observed that a substantial (>30%) increase in total kidney volume correlated with a more severe toxicity grade and the necessity for more aggressive clinical management.

PET-CT imaging unveiled additional insights, showcasing a diffuse augmentation in 18F-fluorodeoxyglucose (FDG) uptake throughout the renal parenchyma. This heightened FDG uptake is a notable radiological indicator in ICI-related nephritis. Moreover, a contrasting reduction in radiotracer activity within the renal pelvis was observed, further highlighting the potential of PET-CT imaging as a valuable tool in the diagnostic arsenal for ICI-induced nephritis.

Ultrasound findings indicated bilateral swelling of renal cortices [9,23]. Despite ultrasound’s widespread availability and cost-effectiveness, there is insufficient evidence to support its utility in diagnosing ICI nephritis. While it can effectively rule out post-renal causes of AKI, its role in specifically diagnosing ICI nephritis remains unclear. In the referenced study, although most patients underwent ultrasound during nephritis, the absence of prior baseline scans hindered the differentiation between abnormalities attributable to nephritis and pre-existing conditions.

The utilization of advanced imaging modalities as a complementary diagnostic approach holds the promise of providing critical guidance for the management of ICI-related nephritis.

### 3.5. Biomarkers for IrAE Nephritis

In the realm of diagnosing ICI-AKI, renal biopsy remains the current gold standard for confirmation. Currently, there is no validated serum biomarker for the diagnosis of ICI-related nephritis in clinical practice. However, there are some biomarkers that are under investigation and hold promise as potential indicators. Notably, a retrospective study highlights the potential of elevated soluble interleukin-2 receptor (sIL-2R) levels as a highly indicative biomarker for identifying ICI-AKI [24]. The application of sIL-2R measurement emerges as a valuable diagnostic aid for ICI-induced renal injury, potentially streamlining the diagnostic process. ICI-related AIN cases have demonstrated increased positivity for PD-L1 and programmed death-ligand 2 (PD-L2) staining compared to other causes of AIN [25] (immunohistochemical staining for these markers can be a valuable adjunct in the diagnostic process).

Furthermore, distinguishing AIN resulting from ICI therapy from other renal conditions can be facilitated by assessing urine biomarkers, specifically tumor necrosis factor-alpha (TNF-α) and interleukin-9 levels. Patients experiencing ICI-related AIN exhibit elevated levels of these biomarkers, offering a non-invasive means of differentiation [26]. This development in urine-based diagnostics can significantly contribute to accurate and timely diagnoses in the context of ICI-AKI.

A recent study has identified elevated levels of serum C-reactive protein (CRP) and a higher ratio of urine retinol-binding protein (URBP) to creatinine in patients with ICI-related AKI compared to those with non-ICI-related AKI [27]. These non-invasive markers emerge as pivotal tools for distinguishing ICI-induced AKI cases. Collectively, the integration of non-invasive diagnostic markers can elevate diagnostic accuracy for ICI-AKI and stimulate the evolution of more effective diagnostic protocols. Further multi-center studies are needed to validate and refine the utility of these biomarkers and integrate them into routine clinical practice for the early and accurate diagnosis of ICI-induced AKI.

### 3.6. Renal Biopsy

The subject of renal biopsy in the context of ICI-related nephritis has sparked divergent perspectives and recommendations. The American Society of Clinical Oncology (ASCO) guidelines advocate a cautious approach, suggesting that renal biopsy may be omitted in favor of the prompt initiation of corticosteroids when an alternative cause of AKI is not readily apparent [28]. This stance is rooted in the belief that immediate treatment may be more critical than histological confirmation in cases where clinical indicators strongly suggest ICI-related nephritis.

In contrast, the National Comprehensive Cancer Network (NCCN) guidelines adopt a more inclusive stance, recommending the consideration of renal biopsy for cases demonstrating Common Terminology Criteria for Adverse Events (CTCAE) grade 2 or higher kidney toxicity [29]. This approach places a greater emphasis on the potential diagnostic value that renal biopsy can provide in terms of elucidating the underlying pathology and guiding treatment decisions. The NCCN’s approach acknowledges the complexities inherent in ICI-related nephritis and underscores the importance of a comprehensive diagnostic assessment.

Meanwhile, the European Society of Medical Oncology (ESMO) guidelines adopt a middle-ground perspective, suggesting that the decision to pursue a renal biopsy should be the result of collaborative discourse with a nephrologist, particularly in cases of grade 2 or higher renal toxicity [30]. This approach seeks to strike a balance between the imperative of early diagnosis and the value of histological insights in optimizing therapeutic strategies.

These varying guidelines underscore the ongoing debate within the medical community regarding the role and timing of renal biopsy in the context of ICI-related nephritis. The diversity of recommendations reflects the complex nature of this condition, with no one-size-fits-all solution. It is a testament to the ongoing research and the need for a case-by-case assessment to determine the most appropriate course of action in diagnosing and managing ICI-related nephritis.

### 3.7. Pathological Features

The histopathological patterns observed in the context of ICI-AKI exhibit notable heterogeneity. Among these patterns, ATIN emerges as the most frequently documented observation in cases of ICI-related AKI. ATIN may manifest either in isolation or in combination with various other glomerular or tubular pathologies [18,31,32]. While reports of ATN have surfaced, it remains a subject of ongoing investigation to determine whether ATN should be unequivocally categorized as an irAE [33,34]. Additionally, a limited number of reports have highlighted the occurrence of renal tubular acidosis in conjunction with ICI therapy [35,36,37].

A systematic review and meta-analysis of biopsy-proven cases and series addressing glomerular pathology associated with ICI treatment have shed light on the diverse spectrum of renal manifestations. This comprehensive analysis reveals that pauci-immune glomerulonephritis and renal vasculitis constitute the most prevalent types, accounting for approximately 27% of cases. Subsequently, podocytopathies, including focal segmental glomerulosclerosis and minimal-change glomerulopathy, collectively make up 24% of the observed pathologies. Furthermore, complement glomerulonephritis contributes to 11% of the cases under scrutiny [38]. Significantly, it is noteworthy that AIN coexists with other renal pathologies in a substantial 41% of instances, further emphasizing the complexity of ICI-related nephritis.

Additionally, there have been documented instances of renal complications associated with vascular involvement, such as thrombotic microangiopathy (TMA) [39] and vasculitis [31,40]. These manifestations underscore the multifaceted nature of ICI-related nephritis and highlight the need for a comprehensive understanding of the varied pathological presentations that can occur in the context of immune-related renal adverse events. The classification of these pathologies has been synthesized based on an extensive review of the existing literature, contributing to our knowledge of the diverse renal manifestations associated with ICI therapy (Table 1).

## 4. Management

In cases where there is an evident rise in creatinine levels, it is prudent to exercise caution and contemplate the temporary suspension of therapy. This pause allows for a comprehensive exploration of potential underlying causes, which may encompass recent intravenous (IV) radiographic contrast administration, states of dehydration, the use of other nephrotoxic medications, including concurrent chemotherapy, or the presence of a urinary tract infection. Careful consideration of these factors is essential in the diagnostic process.

For patients presenting with an elevation in creatinine unattributable to any apparent alternative cause, or those who exhibit an inadequate response to alternative treatment modalities, a presumptive diagnosis of immune-related renal toxicity should be entertained. In such scenarios, empirical treatment becomes a pivotal course of action to address the underlying condition effectively.

In cases of AKI graded as level 2 or level 3, the consensus is that immunotherapy should be temporarily discontinued. Simultaneously, the initiation of steroid therapy is strongly recommended to mitigate immune-related renal toxicity and promote renal recovery.

The prevailing management recommendations for ICI-AKI, as per the guidelines established by the NCCN and ASCO, are concisely summarized in Table 2, providing a comprehensive reference for clinicians to navigate the complexities of ICI-induced renal adverse events [28,29].

In a recent comprehensive meta-analysis, a substantial majority of patients with ICI-AKI, ranging from 82% to 86%, received treatment with corticosteroids. Notably, the administration of corticosteroids commenced at a median of 4 days from the initial diagnosis, and patients continued to receive this therapeutic regimen for a median duration of 41 days. Additionally, a smaller subset of patients, constituting approximately 5% to 9% of the cohort, were managed with alternative immunosuppressive agents [18,32]. The timing of corticosteroid initiation emerged as a significant factor, with early administration (within 3 days of diagnosis) showing a distinct advantage in terms of higher odds of achieving renal recovery when compared to delayed initiation (beyond 3 days).

While the majority of patients exhibit positive responses to steroids alone, those experiencing recurrent AKI or facing challenges in steroid tapering may benefit from alternative immunosuppressants, such as mycophenolate mofetil, infliximab, and azathioprine. A case series involving 10 patients with relapsing ICI-AKI demonstrated a notable 80% achievement of durable complete or partial renal recovery with the TNF antagonist, infliximab [41]. However, further clinical data are imperative to establish optimal dosage and duration recommendations for these immunosuppressants.

Furthermore, the prospect of re-initiating ICI therapy becomes a pertinent consideration in cases where AKI is graded as less severe than level 2 and demonstrates improvement with corticosteroid treatment. There are instances wherein patients have been successfully re-challenged with ICIs even after experiencing grade 3 or 4 renal irAEs, without a recurrence of these deleterious effects [18]. It is noteworthy that this approach may not be universally applicable, especially in patients with glomerular pathologies like systemic vasculitis or nephritic syndrome. In such cases, a more individualized and cautious approach may be preferred to balance the potential benefits and risks of ICI re-challenge in the context of renal-specific irAEs.

## 5. Impact of ICI-AKI

The collective findings from various meta-analyses indicate that renal recovery post-ICI-associated nephritis ranged from 40% to 65%, underscoring that a significant portion of patients may develop CKD as a consequence of these immune-related adverse events. Notably, patients who experienced recurrent AKI following re-challenge with ICIs were observed to face an elevated risk of mortality [32]. It is noteworthy that among those with CKD, individuals in stage three exhibited a notably higher mortality risk when compared to patients in CKD stages one and two. These observations shed light on the importance of vigilance in monitoring renal function and the proactive management of CKD in patients who have undergone ICI therapy.

## 6. Is Nephritis a Predictor of Response?

irAEs have been established as potential markers for predicting the clinical response in patients with non-small cell lung cancer (NSCLC) and melanoma [42,43]. Notably, the irAEs observed in these investigations were primarily associated with skin and endocrine manifestations. However, the extent to which nephritis may serve as a predictive indicator of a favorable treatment response remains uncertain.

A data registry maintained at the University of Texas Southwestern Medical Center focused on patients with renal cell carcinoma (RCC) [41]. Within this context, 36 individuals with metastatic renal cell carcinoma were identified, all of whom exhibited a creatinine concentration exceeding 1.5 times their baseline levels and had received at least one dose of ICI therapy. It is important to acknowledge the limited available data, as only four cases of biopsy-proven ICI-associated nephritis were reviewed in this study. Interestingly, these four patients demonstrated prolonged disease control without the need for systemic therapy, as reported by Patel et al. [44].

A plausible hypothesis that emerges from these observations is the concept of shared epitopes between normal renal epithelial cells and RCC. This hypothesis posits that such shared epitopes may trigger a robust and sustained immune response, potentially contributing to the durable disease control witnessed in these cases.

## 7. Areas for Future Research

The future of ICI-AKI research holds several promising avenues: Firstly, investigating whether ICI-AKI can serve as a predictive marker for positive treatment responses in certain cancers is a compelling direction, as limited data currently exist in this context. Secondly, the integration of non-invasive diagnostic tools, such as biomarkers and advanced imaging techniques, holds substantial promise for enhancing early detection and management, with a need for validation and clinical integration. Thirdly, a deeper understanding of the risk factors and underlying mechanisms behind ICI-related nephritis is essential to optimize treatment strategies. This includes exploring auto-reactive T cells, auto-antibodies, and other contributing factors to devise new therapeutic approaches. Fourthly, refining treatment protocols and developing consensus recommendations, particularly concerning the use of renal biopsies and re-initiation of ICI therapy, can improve patient care. Finally, assessing renal recovery, the risk of chronic kidney disease, and the correlation between recurrent AKI and mortality in ICI-AKI patients is vital for understanding long-term outcomes and further advancing the field of cancer immunotherapy.

## 8. Conclusions

In conclusion, the advent of ICIs has transformed cancer therapy, showing promise in treating various malignancies. However, challenges, particularly the emergence of irAEs like ICI-related nephritis, are noteworthy. Investigating the intricate molecular mechanisms reveals multiple potential causes, emphasizing the complexity of this condition. Early diagnosis and risk assessment are crucial for effective management, as ICI-related nephritis, while not highly incident, can lead to severe consequences. Identifying high-risk patients and utilizing diagnostic tools, including non-invasive biomarkers, is essential. The gold standard for diagnosis, renal biopsy, remains a topic of debate. Management involves corticosteroids, with considerations for immunosuppressive agents in refractory cases. Cautious re-initiation of ICI therapy is possible in select cases. Renal recovery rates and long-term outcomes are crucial considerations. The association between irAEs and favorable responses in specific cancers warrants further exploration, potentially impacting treatment strategies. In summary, while ICIs revolutionize cancer treatment, addressing irAEs like ICI-related nephritis requires continuous research and refined approaches for successful therapy.

## Figures and Tables

**Table 1:** Pathologic features of ICI-AKI.

Glomerular Compartment	Tubulo-Interstitial Compartment	Vascular Compartment
Nephrotic syndrome Minimal change disease Focal segmental glomerulosclerosis (FSGS) Membranous glomerulopathy Amyloidosis	Acute tubulointerstitial nephritis (ATIN) Acute tubular necrosis (ATN) Renal tubular acidosis (RTA)	Renal vasculitis Thrombotic microangiopathy (TMA)
Non-nephrotic/nephritic Complement 3 glomerulonephritis (C3GN) Lupus IgA Anti-glomerular basement membrane antibody (GBM) Pauci-immune		

**Table 2:** Grading of ICI-nephritis and current recommendations on management.

Grade, per NCI (National Cancer Institute) CTCAE v5.0 Acute Kidney Injury	Definition	Initial Management	Follow Up
Grade 1	Creatinine increased 1.5–<2× baseline or increase of ≥0.3 mg/dL over 48 h	Consider holding immunotherapy	If improved to baseline, continue monitoring
Grade 2	Creatinine increased 2–<3× baseline	Prednisone 0.5–1 mg/kg/day	If improved to grade 1: taper steroids over 4 weeks. If persistent: treat as grade 3
Grade 3	Creatinine increased ≥3.0× baseline; 4.0 mg/dL	Prednisone/IV methylprednisolone 1–2 mg/kg/day	If improved to grade 1, taper steroids over 4 weeks. If kidney injury remains >Stage 2 after 4–6 weeks of steroids or if creatinine increases during steroid taper (or once off steroids) (in alphabetical order): Azathioprine Infliximab Mycophenolate
Grade 4	Life-threatening consequences, need for renal replacement therapy (RRT); dialysis as indicated	Prednisone/IV methylprednisolone 1–2 mg/kg/day	If improved to grade 1, taper steroids over 4 weeks. If kidney injury remains >Stage 2 after 4–6 weeks of steroids or if creatinine increases during steroid taper (or once off steroids) (in alphabetical order): Azathioprine Infliximab Mycophenolate

## Data Availability

No new data were created or analyzed in this study. Data sharing is not applicable to this article.
